# Teaching Mathematical Modelling and Programming with GAMS in Dual Management Master Curricula Using Flipped Classrooms and Open Book Exams

**DOI:** 10.1007/s43069-022-00162-8

**Published:** 2022-08-27

**Authors:** Michael Jahr

**Affiliations:** European University of Applied Sciences, Neusser Str. 99, 50670 Cologne, Germany

**Keywords:** Teaching OR, Flipped classroom, Open book exam, Dual curricula studies

## Abstract

In this article, we present a flipped classroom based teaching concept and related open book exam for Master’s courses in the field of Operational Research while paying particular attention to dual curricula management students. The characteristics of dual curricula include that students study part-time complemented by practical stages in cooperating firms. As a consequence, there is a limited number of lectures available compared to full-time programs. Hence, adequate course structures are needed to facilitate learning and encourage students to explore the research field further. We present an illustrative teaching concept focusing on the well-known Resource Constrained Project Scheduling Problem (RCPSP) and GAMS programming. Moreover, we demonstrate how self-study phases and classroom trainings are systematically combined to support the management students’ autonomous programming activities culminating in 24-h open book exams.

## Introduction

For young business professionals, especially in the so-called generations “y” and “z” [1], digitalization and sustainability will be the enduring hot topics of their career, accompanied by the macrosocial issue of healthcare [2, 3]. The latter has not least received special attention in the academic discussion of the COVID-19 pandemic in 2020/2021. It can be assumed that there is a strong positive correlation between these three topics, that is, digitalization will serve as a driving force to achieve the sustainability and health goals [3, 4]. Therefore, it is decisive for the individual educational careers that digitalization is thoroughly considered in higher education. In this regard, a thorough consideration of topics such as digitalization and machine learning or artificial intelligence can be achieved by dealing with mathematical programming and coding [5, 6]. This is particularly challenging for dual-curricula management students. These students study part-time complemented by practical stages in cooperating firms, thus reducing the number of actual courses of higher education. Furthermore, dual curricula management studies, especially on the Bachelor’s level, mainly focus on qualitative subjects, e.g. organization, marketing, and leading [7]. The (dual) management graduates are expected to coordinate resources and make decisions rather on qualitative level than on quantitative level. Hence, in-depth consideration of program code and the underlying modelling is mostly outsourced to specialists, e.g., computer scientist. The fact that, in management curricula, the operational research-related courses are only taken into account to a limited extend is an aggravating factor. Consequently, these graduates only have insight into the operational research methodology. Certainly, our experiences from various universities and study programs show that graduates from full-time Bachelor’s programs in general do not have significantly higher knowledge. Not to mention the general reservations regarding quantitative methodology or mathematics [8]. Therefore, for teachers in consecutive Business Master’s courses, it can be difficult to create uniform levels of knowledge or an advanced level. However, mathematical modelling and programming requires competencies in abstract thinking, which is acquired through continuous practicing. Thereby, we see that due to the growing relevance of digitalization, this is not so much a question of motivation, but rather a question of course structure and guidance. In this context, we would like to share our positive experiences using the flipped classroom concept together with algebraic modelling software, i.e., GAMS. The remainder of this article is structured as follows. First, we present the didactical concept of a sample Business Master’s course in Operational Research that we teach at several universities in international study programs with predominantly logistics and business development focus. We also highlight specific requisites with regard to dual curricula students and open book exams that we also successfully used during the compulsorily online teaching phases in 2020/2021 due to the COVID-19 pandemic. Then, we discuss a sample GAMS program code and related didactical aspects. Finally, some experiences are given regarding how to motivate management students to further deal with programming.

## A Flipped Classroom Teaching Unit Concept for the Resource Constrained Project Scheduling Problem

**Fig. 1:**
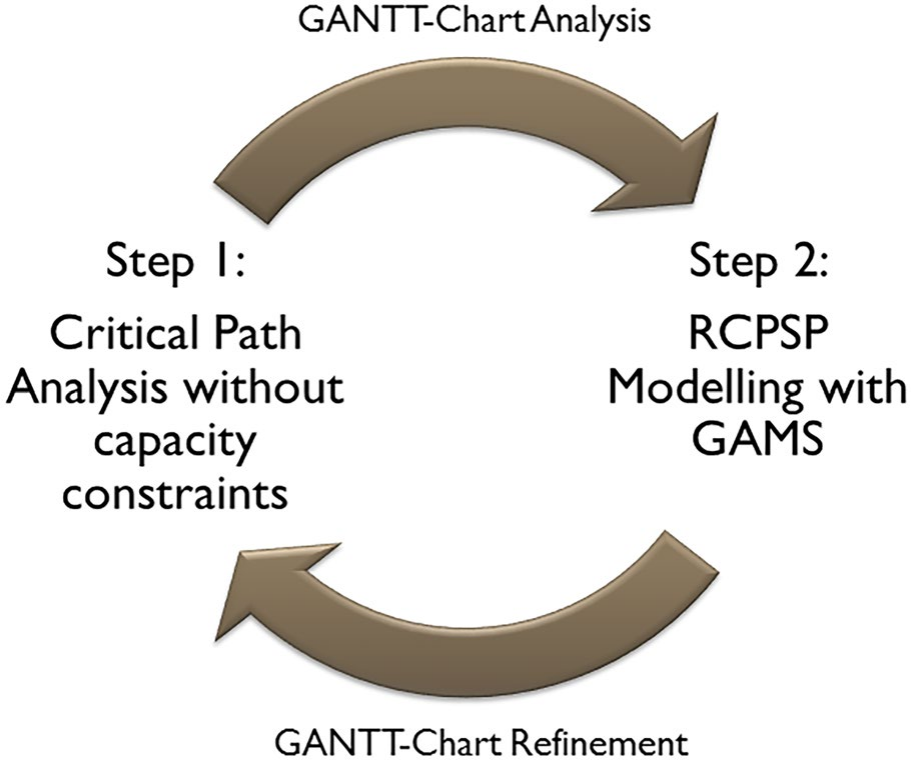
Teaching approach for the RCPSP

We consider a dual curricula Master’s study program in the field of business development and therefore a classical management program with no explicit focus on mathematical programming but rather on marketing, organization, and strategic management. Here, students study part-time in addition to their regular jobs. The curriculum is integrated into the working time, so lectures take place on specific reserved weekdays and weekends during the semester. For the considered Master’s course dealing with applied operational research methods in various management fields, we take into account a total workload of 180 academic hours of 45 min each. The total workload is divided into 60 contact hours for 15 weeks, i.e., 4 semester periods per week, and 120 h self-study that we use for homework, pre-, and post-preparatory tasks. Therefore, we can use 4 contact hours and 8 self-study hours per week for a topic (see Table 1). As course contend, we use basic models and further model extensions from strategic, tactical, and operational business issues. For example, from our perspective one of the most useful operational models to highlight the benefits of programming is the Resource Constraint Project Scheduling Problem (RCPSP), as it combines rather complex modelling with excellent practical transferability [9, 10]. Thus, students can interconnect the practical task of managing real-life projects and the transfer to an automated sequencing approach via mathematical programming. Furthermore, students can create back-references from the modelling to practical issues, because the practical application of the RCPSP, as stated below, requires a two-step approach (see Fig. 1). Next to project scheduling we also integrate strategic approaches into the course content, e.g., location planning, tactical models, e.g., lot-sizing with the (ML) CLSP [11], and related heuristics [12]. For the different topics, our didactic approach is standardized, so that we focus on the RCPSP as a prime example. Here, we specify a workload of 24 academic hours for the RCPSP, which is slightly more than one-eighth of the available total course workload (see Table 1).

**Table 1:** Two weeks teaching unit

*Lecture week*	*Pre-preparatory* *self-study* *4 h*	*Class work* *4 contact hours*	*Post-preparatory* *self-study* *4 h*
Lecture week i	Literature and course material studies. i.e., Brucker and Drexl (1999)	Case study 1 and code programming standard model	Autonomous programming and analysis of model extension 1
Lecture week i + 1	Autonomous programming and analysis of model extension 2	Review of model extensions 1 and 2 Case study 2 with model extension 3	Test questions on online learning platform, e.g. Moodle or ILIAS

As shown in Fig. 1 first, the students must perform a critical path analysis disregarding limited resource capacities based on Activity-on-Node (AoN) [13, 14] for a specific project, e.g., IT-project or product development [15]. Hence, a project lead time can be calculated creating infeasible solutions due to exceeded resource capacities. Second, using Gantt charts and manual activity shifts, feasible solutions are created that in general result in longer project lead-times [16]. Moreover, capacity investments can be discussed as alternative to meet the shorter lead-time. However, at the second step, the RCPSP is applied to automate the activity shifting and to create feasible optimal solutions. Thus, we use Gantt charts for a simple explanation of the model function and its structure. The learning aspect is that students can quickly understand the model mechanism and connect it to a common practical problem, i.e., project management with deadlines (and limited budgets).

The basic concept of flipped classrooms is that students must acquire subject content individually, so classes are reserved for case studies, practical exercises, and joint programming, which generally has a positive impact on learning [17, 18]. In contrast, in classical classrooms teachers deal with content work and provide students with extra tasks, e.g., as homework. Yet, for complex mathematical programming, there must be a balanced course structure [19]. Therefore, in the first week, we provide reading material and digital content, i.e., teaching videos or simple interactive completion tests for self-evaluation, and the first case study as preparatory stage. Case study 1 represents a standard project, e.g., irregular IT-projects . We ask the students to prepare an AoN, followed by a critical path analysis and a Gantt chart of the resulting sequence. This should be standard knowledge from Bachelor’s courses and thus is not discussed separately as specific course contend. Then, in the first classroom training, we deal with the mathematical model and its implementation in GAMS (see Chapter 3). We discuss the standard version with just renewable resources and assign the autonomous preparation of model extensions as task. First, we use simple extensions of parameters, e.g., additional resources and/or project tasks. Subsequently, in the post-preparatory stage, the students can compare their solutions with uploaded online samples. Next, for the second pre-preparation stage, we provide a task to implement a more complex model extension, i.e., the integration of non-renewable resources (budgets). In the following second classroom training, there is a short review of the first two model extensions and the programming of a new case study 2 dealing with a different area of application, e.g., product development or consulting projects. We supplement the second case with a final model extension, that is, multiple non-linear objectives or multiple parallelized projects or activity splitting and preemption [10, 20–24]. Finally, we provide additional learning functions on the digital platforms for the second post-preparatory stage, i.e., online tests, forums, and chat function. This basic structure is consistently repeated throughout the course with different models from various business areas, which are compiled along a common thread connected to the overall study program goals, i.e., business development techniques. In this way, we create continuous programming and modeling activities for the management students. Finally, we chose the open book as an adequate test type [25]. Basically, we used open book exams or similar examination formats already prior to the compulsorily online teaching using Zoom or MS Teams due to the COVID-19 pandemic. This type of exam corresponded well to the flipped classroom principle. Especially, we had good experiences with one-day exam periods, for example, from 9 to 9 am the following day. Hence, we provide a case study as audit task similar to the examples from the course. The examinees must program the source code in GAMS, visualize, and analyze the results in AoN and Gantt charts. For example, in the project scheduling case, the examinees must create an optimal project plan. Moreover, on the Master’s level, we also expect that students solve smaller transfer tasks that were not explicitly discussed in the course, e.g., data search and comparison in an organizational context. Our experience shows that this type of exam offers students more space for individual solutions, making use of their professional experiences, which is a central element in dual curricula study programs. A sample open book exam for the RCPSP is formulated as follows:

Allowed aids:

No restrictions.

Evaluation Guidelines:

Your solution will be evaluated like an essay. Therefore, the evaluation criteria include the structure, the line of arguments, transparency, consistency, correctness, and the combination of the tasks.

Notes:

Please create one single file (preferably in PDF format) for your solution. Do not use more than 2 pages for the solution of each task using 1.5 line spacing. Scans of handwritten solutions are allowed. You may include screenshots.

Task (100 points):

For an IT-project, two internal employees are available. You take on the role of the project manager without performing operational tasks. Create a feasible project plan indicating the GoLive of the considered system integration. The following project tasks have been identified:

**Table:** 

*Task*	*Duration* *(days)*	*Resource requirements* *(personnel)*
*A: Kick-off meeting*	*1*	*2*
*B: Technical coordination*	*2*	*2*
*C: System architecture*	*1*	*1*
*D: Data structure*	*1*	*1*
*E: Coordination with specialist department*	*2*	*2*
*F: Documentation*	*1*	*1*
*G: Back- and frontend programming*	*5*	*2*
*H: Training*	***?***	*1*
*I: Test scenarios*	*1*	*1*
*J: Bug Fixing*	***?***	*2*

It is clear that task A must be performed before tasks B and C. Then, task B must be finished before task D and E. Moreover, task C is the predecessor of tasks F and G. Also, task D must be finished before task G. Task E is a predecessor of tasks H and I. Finally, task J is the successor of tasks F, G, H and I.

- *Complement missing data in the project list. Justify your statements.*
- *Create the AoN.*
- *Perform a critical path analysis.*
- *Indicate resource conflicts based on a Gantt chart.*
- *Create an optimal project plan using GAMS and visualize the results in the Gantt chart.*

## Implementing the RCPSP in GAMS as Teaching Content

In Sect. 2, we described our teaching concept for mathematical modelling based on flipped classroom. Now, we highlight the specific implementation of the considered RCPSP with GAMS. For a start, we outsource the mathematical modelling to the first pre-preparatory self-study stage. Here, we use the standard formulation as shown, for example, by Brucker and Drexl [9]:

## Symbols

${\mathrm{Capacity}}_{\mathrm{r},\mathrm{t}}$ – Available renewable capacity of resource *r* in period *t.*

$${\mathrm{d}}_{\mathrm{j}}$$ – Job duration.

${\mathrm{EST}}_{\mathrm{j}}$ – Earliest starting time for job *j.*

${\mathrm{LST}}_{\mathrm{j}}$ – Latest starting time for job *j.*

$\mathrm{j}\in \mathrm{J}$ – Jobs index and $\left(\mathrm{h},\mathrm{j}\right)\in {\mathrm{P}}_{j}$ precedence relation of jobs *h* and *j.*

R – Renewable resources index.

T – Time index.

${\mathrm{tb}}_{\mathrm{j},\mathrm{r}}$ – Capacity utilization coefficient for job $\mathrm{j }\in \mathrm{J}$ performed by resource $\mathrm{r }\in \mathrm{R}$

${\mathrm{x}}_{\mathrm{j},\mathrm{t}}$ – Binary variable indicating the job completion time.

$$\mathrm{Z}$$ – Project makespan

1 $$\underset{{x}_{j,t}}{\mathrm{Min}}\mathrm{Z}={\sum }_{\mathrm{t}={EST}_{J}}^{{LST}_{J}}t\cdot {x}_{J,t}$$

2 $${\sum }_{\mathrm{t}={EST}_{j}}^{{LST}_{j}}{\mathrm{x}}_{\mathrm{j},\mathrm{t}}=1 \forall \mathrm{j\epsilon J}$$

3 $${\sum }_{\mathrm{j}=1}^{\mathrm{J}}{\mathrm{tb}}_{\mathrm{j},\mathrm{r}}\cdot {\sum }_{\mathrm{q}=\mathrm{max}\left\{\mathrm{t},{\mathrm{EST}}_{\mathrm{j}}\right\}}^{\mathrm{min}\left\{\mathrm{t}+{\mathrm{d}}_{\mathrm{j}}-1,{\mathrm{LST}}_{\mathrm{j}}\right\}}{\mathrm{x}}_{\mathrm{j},\mathrm{q}}\le {\mathrm{Capacity}}_{\mathrm{r},\mathrm{t}} \forall \mathrm{r\epsilon R},\forall \mathrm{t\epsilon T}$$

4 $${\sum }_{\mathrm{t}={EST}_{h}}^{{LST}_{h}}\mathrm{t}\cdot {\mathrm{x}}_{\mathrm{h},\mathrm{t}}\le {\sum }_{\mathrm{t}={EST}_{j}}^{{LST}_{j}}\left(t-{d}_{j}\right)\cdot {\mathrm{x}}_{\mathrm{j},\mathrm{t}} \forall \mathrm{j\epsilon J},\forall \mathrm{h\epsilon }{P}_{j}$$

5 $${x}_{\mathrm{j},\mathrm{t}}\upepsilon \left\{\mathrm{0,1}\right\} \forall \mathrm{j\epsilon J},\forall \mathrm{t\epsilon T}$$

From a didactical perspective, the binary decision variable definition ${x}_{j,t}$ as indicator for activity completion times is often a difficulty for beginners, because the usual idea of projects is chronological. Therefore, teachers must ensure that students understand the definition. This can be facilitated by Gantt charts in which project tasks are sketched in backwards starting from the task completion times. Whereas capacity constraints (3) are often familiar to Master’s students, the sequence constraints (4) are sometimes unfamiliar. It is hence important that students combine the modelling with an AoN, so that a visual representation of predecessor-successor relations is available. Again, the overall model function can be well explained in a Gantt chart by shifting tasks along the time axis to change capacity usages and thus creating feasible sequences. This also shows that the model is an automation approach for a task that can be performed manually. In the first classroom training, our focus is on programming and testing the GAMS source code of the RCPSP. We use the following source code with some sample data for the standard version:

*$OnText*

Source Code for the Resource Constrained Project Scheduling Problem.

*$Offtext*

*Set of Activities, Resources and Project Horizon

set

J Activities /A, B, C, D, E, F, G, H, I/

R Resources /r1/

T TimeFrame /t1*T30/;

*Re-Definition of set symbols

alias (h,j);

alias (t,q);

*Activity-on-Node

set

path(h,j) /A.B, A.C, B.D, C.D, C.E, C.F, D.I, E.G, E.H, F.H, G.I, H.I/;

*Definition of model variables

variable

Makespan;

binary variable

x(j,t);

*Project data

parameters

*Critical Path Analysis

EST(j) /A 3, B 7, C 5, D 13, E 10, F 8, G 17, H 15, I 25/

LST(j) /A 8, B 16, C 10, D 22, E 15, F 17, G 22, H 22, I 30/

*Activity times

d(j) /A 3, B 4, C 2, D 6, E 5, F 3, G 7, H 5, I 8/

*Renewable capacity

Capacity(r,t) /

r1.t1 2,r1.t2 2,r1.t3 2,r1.t4 2,r1.t5 2,r1.t6 2,r1.t7 2,r1.t8 2,r1.t9 2,

r1.t10 2,r1.t11 2,r1.t12 2,r1.t13 2,r1.t14 2,r1.t15 2,r1.t16 2,r1.t17 2,

r1.t18 2,r1.t19 2,r1.t20 2,r1.t21 2,r1.t22 2,r1.t23 2,r1.t24 2,r1.t25 2,

r1.t26 2,r1.t27 2,r1.t28 2,r1.t29 2,r1.t30 2/

*Renewable capacity usage

tb(j,r)/ A.r1 1, B.r1 1, C.r1 1, D.r1 1, E.r1 1, F.r1 1, G.r1 1, H.r1 1, I.r1 1/;

*Definition of equations

equations

objective

CO1(j), CO2(r,t), CO3(h,j);

*Mathematical Model

objective.. Makespan = E = sum((t)$(ord(t) ge EST('I') and ord(t) le LST('I')),ord(t)*x('I',t));

CO1(j).. sum((t)$(ord(t) ge EST(j) and ord(t) le LST (j)), x(j,t)) = E = 1;

CO2(r,t).. sum((j), tb(j,r)*sum((q)$(ord(q) ge max(ord(t), EST(j))and ord(q) le min(ord(t) + d(j)-1,

LST(j))), x(j,q))) =L= Capacity(r,t);

CO3(h,j)$path(h,j).. sum((t)$(ord(t) ge EST(h) and ord(t) le LST(h)), ord(t)*x(h,t)) = L = 

sum((t)$(ord(t) ge EST(j) and ord(t) le LST(j)), (ord(t)-d(j))*x(j,t));

*Solving statements

model Modell /all/;

option mip = cplex;

solve Modell using mip minimizing Makespan;

For beginners, we start with hard-coded inputs to describe the structure of the GAMS modeling environment. One fact that we highlight is the separation of the visible modelling level (frontend) and the invisible solving level (backend). The reason for this is twofold. First, it demonstrates the flexibility of the computing environment compared, for example, to standard Enterprise Resource Planning Systems. These mostly offer only limited variants of specific problems [26]. In contrast, GAMS and similar software environments can be used to program any problem structure and problem-solving approach. Second, the software structure shows that researchers can separate the problem definition from the problem-solving for a better understanding. Also, it is possible to discuss different problem-solving approaches. We prefer GAMS due to its rather simple syntax and easy to understand debugging capabilities. For most of the management students, this is the first time they have to deal with explicit source code, as in Bachelor’s courses in principle linear problems are discussed solvable with spreadsheet applications, i.e., Excel. Thus, GAMS needs only a small number of command lines for the definition of variables and parameters. Furthermore, the model equations can be programmed intuitively compared to the formal mathematical model. In this respect, the considered source code for the RCPSPS already represents one of the more complex variants in our courses. Nevertheless, it is natural for beginners to repeatedly produce errors in their attempts to create working source code. Therefore, we see that GAMS provides easy-to-understand error statements that provide good user guidance for troubleshooting. At the same time, the debugging is an additional didactic element for further model discussion as it raises various student questions. Alternative software, for example, R, showed to be more difficult to learn for beginners. The syntax is less intuitive and more sensitive to coding errors. However, only in the following step, we consider the programming of interfaces to databases in GAMS, e.g., simple Excel file or csv files, via calling the GXXRW function to improve the data management. In courses for dual-curricula management students, this is where we often draw the line. Clearly, data management via interfaces is of practical relevance. However, we want to ensure that management students concentrate on the basic mathematical model and that they understand the complexity of programming being a considerably creative process [27]. Therefore, we want to train efficient professional communication skills, so that future managers can cooperate with IT specialists by learning to create working source code. As a consequence, we keep the programming on a “digestible” level. Nevertheless, in courses for business informatics, we naturally proceed to advanced techniques. For example, we outsource the data management by programming interfaces with dynamic set formulations:

*Excel-input statement with dynamic sets and.txt file usage

**$onecho** > input.txt.

dset = i rng = Excel!A2 rdim = 1.

dset = j rng = Excel!Q1 rdim = 1.

par = d rng = Excel!Q1 rdim = 1.

par = u rng = Excel!A1 rdim = 1 cdim = 1.

*GDXXRW Input Call

**$CALL** GDXXRW C:\users\desktop\Excel_input.xlsx o=C:\users\desktop\Excel_input.gdx trace=3 @input.txt

**$gdxin** C:\users\desktop\Excel_input.gdx

**$load** variables

**$load** parameters

**$gdxin**

*Excel output statement

**execute_unload** "C:\users\desktop\Excel_results.gdx" variables;

**execute** 'gdxxrw.exe C:\users\desktop\Excel_results.gdx

o= C:\users\desktop\Excel_results.xlsx variables;

We see that this code is more technical and exceeds the normal business management scope. For example, R in general provides an easier access to excel interfaces, but at the expenses of the more complex syntax compared to GAMS. In courses for business informatics students, we therefore highlight user friendliness of digitalization, as managers in practice are used to work with spreadsheet-based tools. Another important aspect we discuss in detail is the integration of comments in the source code, which is essential for third parties to understand the code. Furthermore, comments are our didactical link to the mathematical model.

## Concluding Remarks on Creating New Mindsets for Programming Lectures

Based on our experience from many years of teaching operational research and programming, we see that it is not so much a question of setting the relevance of this topic for management students [5, 28]. This question raised every now and then within discussion with other professorial colleagues from different disciplines due to the partially abstract mathematical nature. Often, colleagues argue that management students should maintain a comprehensive overview of businesses rather than dealing with mathematical details. These are maybe more relevant for IT specialists. In fact, management students show great interest in the potential of mathematical programming. However, for them, especially in dual curricula programs, there is a kind of “barrier” between mathematical models and the practical problems, which is difficult to overcome. There is a desire (sometimes even an expectation) for perfect and simple customized solutions for specific complex problems that text book models or scientific articles cannot satisfy. From our perspective, this also explains many communication problems between IT and business departments. For experts, this imbalance is absolutely clear, but for students (and many professionals), it is often a decisive source of frustration [29]. Therefore, a core element of our teaching is to uncover that the mathematical programming and the real-life system elements must approach each other. It is not promising to expect that programming, which is bound to its own logic, can solve a random practical problem without changing the real-life scenario. This is in some way like trying to have cake and eat it, too. Therefore, to make clear that there is a decisive interplay between mathematical programming and the real-life problem can break this barrier in the management students. Then, they begin to understand that dealing with actual programming and program code allows for deeper insights into the considered business processes. The strict mathematical logic of programming reveals inconsistencies in real-life processes and vice versa shows the limits of automating process steps with human interaction. Thus, programming becomes an analysis technique next to a solution or automation approach and offers a great link to knowledge gained in other disciplines. We were often pleased to see that once this context was realized, dual curricula management students autonomously began to dive deeper into codes in GAMS or other software packages like R or AMPL or LINGO and the program logic in order to scrutinize existing processes in their companies.

## Data Availability

The author confirms that the data and source codes supporting the findings of this study are available in the article.
